# The effects of training by virtual reality or gym ball on pelvic floor muscle strength in postmenopausal women: a randomized controlled trial

**DOI:** 10.1590/bjpt-rbf.2014.0148

**Published:** 2016-03-22

**Authors:** Natalia M. Martinho, Valéria R. Silva, Joseane Marques, Leonardo C. Carvalho, Denise H. Iunes, Simone Botelho

**Affiliations:** 1 Curso de Fisioterapia, Escola de Enfermagem, Universidade Federal de Alfenas (UNIFAL-MG), Alfenas, MG, Brazil; 2 Departamento de Cirurgia, Faculdade de Ciências Médicas, Universidade Estadual de Campinas (UNICAMP), Campinas, SP, Brazil

**Keywords:** physical therapy, menopause, muscle strength dynamometer, pelvic floor, virtual reality exposure therapy

## Abstract

**Objective:**

To evaluate the effectiveness of abdominopelvic training by virtual reality compared to pelvic floor muscle training (PFMT) using a gym ball (a previously tested and efficient protocol) on postmenopausal women’s pelvic floor muscle (PFM) strength.

**Method:**

A randomized controlled trial was conducted with 60 postmenopausal women, randomly allocated into two groups: Abdominopelvic training by virtual reality – APT_VR (n=30) and PFMT using a gym ball – PFMT_GB (n=30). Both types of training were supervised by the same physical therapist, during 10 sessions each, for 30 minutes. The participants’ PFM strength was evaluated by digital palpation and vaginal dynamometry, considering three different parameters: *maximum strength, average strength* and *endurance*. An intention-to-treat approach was used to analyze the participants according to original groups.

**Results:**

No significant between-group differences were observed in most analyzed parameters. The outcome endurance was higher in the APT_VR group (p=0.003; effect size=0.89; mean difference=1.37; 95% CI=0.46 to 2.28).

**Conclusion:**

Both protocols have improved the overall PFM strength, suggesting that both are equally beneficial and can be used in clinical practice. Muscle endurance was higher in patients who trained using virtual reality.

## Bullet points

- Clinical practice lacks options for pelvic floor muscle training.
- The gym ball is commonly used in urogynecologic rehabilitation.
- Virtual reality is an innovative method of pelvic floor muscle training.
- Both protocols led to improvement in pelvic floor muscle strength.
- Both protocols can be useful tools in clinical research.

## Introduction

During the postmenopausal period, important physiological changes are observed in women, among them, pelvic floor muscle (PFM) dysfunction^1^. There is evidence that pelvic floor muscle training (PFMT) should be offered as first-line conservative therapy to women with urinary incontinence (stress, urge, or mixed). Strengthening the PFM aims to improve urethral closure pressure, suppress urgency, and promote greater support for the pelvic organs^2^. However, little is known about the effects of training on PFM strength in postmenopausal women^3^.

There has always been an increasing need for protocols that use exercises that improve PFM strength, with the consequent restoration of its function, through enjoyable, stimulating, and appropriate therapy sessions that fit in these women’s condition.

Marques et al.^4^ developed a PFMT protocol using a gym ball and found its efficacy for increasing PFM electrical activity and decreasing urinary symptoms in pregnant and postpartum women. Since then, this protocol has been tested in different populations^5^, demonstrating its effectiveness also for improving anterior pelvic organ prolapse in postmenopausal women^6^. This protocol stimulates repeated PFM maximal contractions, similar to other studies that showed a good methodology quality confirming the effectiveness of PFMT^7^.

Virtual reality has been used in clinical practice in order to explore the game environment while receiving treatment, adding innovation and interactivity to physical therapy routine. Elliott et al.^8^ found positive results related to the feasibility, effectiveness, and participant appreciation of the combined intervention: adding virtual reality to PFMT in elderly women with mixed urinary incontinence. Moreover, they highlighted the need for new randomized controlled trials.

Therefore, the aim of this study was to evaluate the effectiveness of abdominopelvic training by virtual reality compared to pelvic floor muscle training (PFMT) using a gym ball (a previously tested and efficient protocol) on postmenopausal women’s PFM strength.

## Method

### Design, setting and participants

A prospective randomized controlled trial was conducted (Clinical trial no.: RBR-8tsrb7) from July 2012 to November 2013. Women aged over 50 years and in their postmenopausal phase for at least one year or more were included in this study. The participants were selected from the university extension project called *Attention to Women’s Health*, which promotes women’s health activities for patients in the public health system in the city of Alfenas, MG, Brazil, and was approved by the Universidade Federal de Alfenas (UNIFAL), Alfenas, MG, Brazil (PREAE no. 2026).

The study received ethical approval from the Regional Ethics Review Board of the UNIFAL (protocol: CAEE 0306.0.213.000-07), and all participants gave their informed and written consent according to the Helsinki Declaration prior to the initial assessment.

### Exclusion criteria

The exclusion criteria were: urinary tract infection, myopathy, neurological abnormalities, diseases which have a collagen alteration, cognitive and physical disorders that would hinder participation in either evaluation or training programs, any pelvic organ prolapse greater than or equal to three on the Pelvic Organ Prolapse Quantification (POP-Q) system, PFM strength grade zero on the Modified Oxford Grading Scale^9^, and previous PFMT supervised by health professionals. Women undergoing hormone replacement therapy were included as long as their prescriptions had been stable for at least six months^10^.

### Randomization

According to the inclusion and exclusion criteria, 60 postmenopausal women were randomly assigned into two training groups through a simple randomization schedule (using computerized random numbers): Abdominopelvic training by virtual reality (APT_VR) and Pelvic floor muscle training using a gym ball (PFMT_GB). The allocation of the subjects was concealed by using sequentially numbered, sealed, opaque envelopes. On the first day of treatment, the envelope allocated to the participant was opened by the physical therapist who provided the training. Each participant was aware of the possibility of participating in one group or the other.

**Figure 1:**
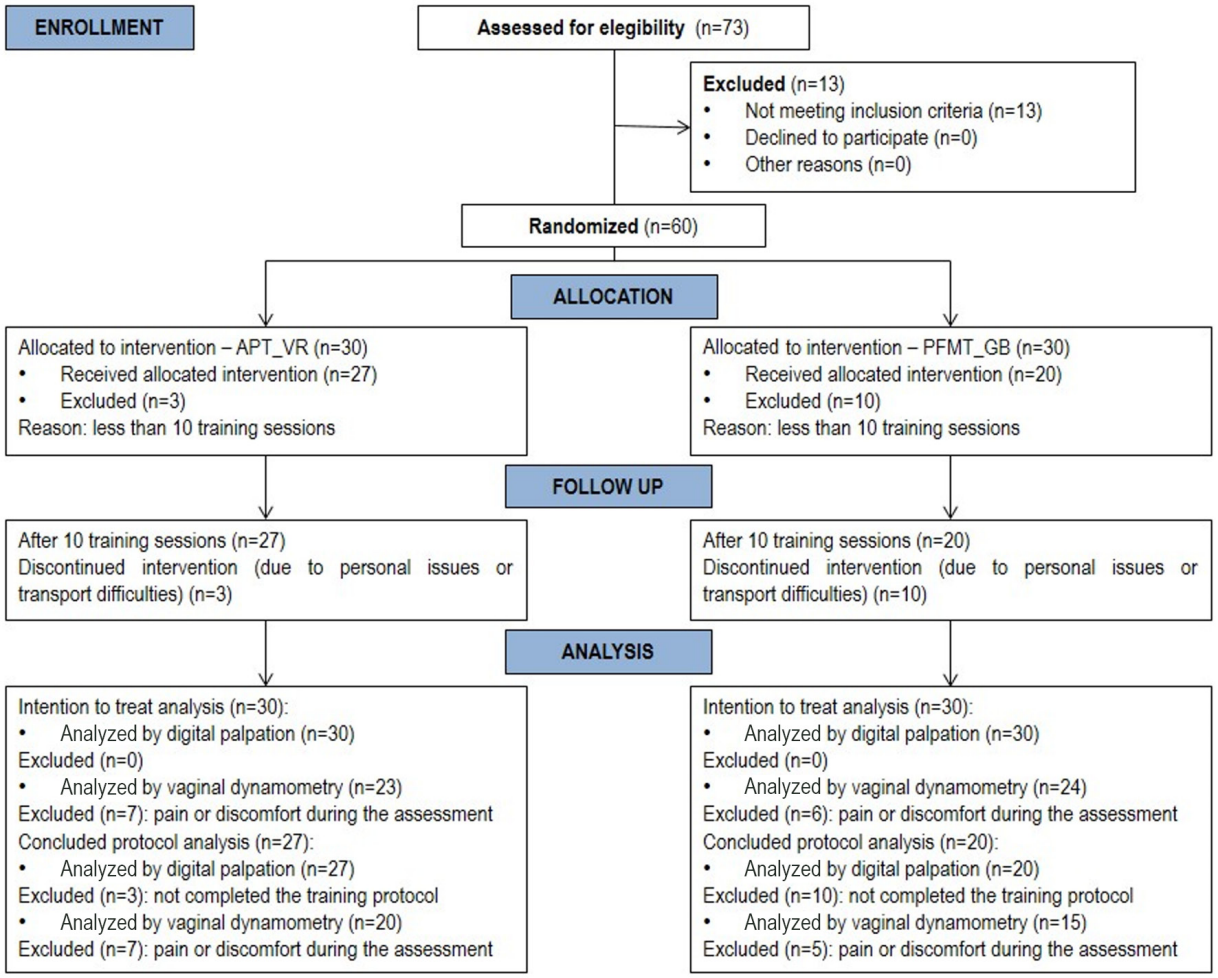
Studied population. APT_VR: Abdominopelvic training by virtual reality; PFMT_GB: Pelvic floor muscle training using a gym ball.

Of the 60 participants initially included in the study, 47 completed the protocols: 27 from the APT_VR group and 20 from the PFMT_GB group (Figure 1).

To reduce the risk of contact among the participants of the two groups, the interventions were carried out on different days.

### Outcome measure: pelvic floor muscle assessment

First, the participants were asked to give their demographic and clinical data, then a physical evaluation was performed, which consisted of assessing PFM strength using two tests: 1) digital palpation (secondary outcome), which is a clinical, subjective, and functional test, commonly used in clinical practice; and 2) vaginal dynamometry (primary outcome), which is an objective method for measuring PFM strength. This assessment was carried out before and five weeks after the protocols by the same evaluator, who has comprehensive knowledge and experience in PFM assessment skills. The data analysis was performed by a second researcher, who did not accompany the assessment and treatment processes.

The participant was placed in supine position with lower limbs flexed and feet on the stretcher. The PFM evaluation was then conducted, starting with digital palpation followed by vaginal dynamometry so that the subjective estimation of the former would not be affected by the objective results of the latter^11^.

### Digital palpation

During digital palpation, the examiner introduced the index and middle fingers, 2-3 cm into the vaginal opening, performing an abduction movement, while the patient was asked to perform a maximum contraction of the muscles, lifting inward and squeezing around the fingers^12^. Muscle strength was graded on the Modified Oxford Grading Scale (0-5 points)^9^.

Next, the participants were taught how to correctly contract the pelvic floor and transversus abdominis (TrA) muscles without using other accessory muscles or performing inspiratory apnea or Valsalva maneuver, in order to be able to maintain a correct muscle performance throughout the intervention.

### Vaginal dynamometry

As an objective evaluation of PFM strength, a vaginal dynamometer (EMG System do Brasil, model DFV 020101/10^®^) was used. It is a cylindrical-shaped device 9.5cm in length and 3.3cm in diameter, without any opening adjustment options, equipped with a load cell 2 cm from the base that can measure anteroposterior unidirectional compressive strength in kilogram/force (Kgf) units (1Kgf=9.8 *Newton*). The vaginal dynamometer was connected to a computer and both remained unplugged from the mains during collections to avoid any interference.

The vaginal dynamometer was covered with a condom (*Elite* ^®^), lubricated with hypo-allergenic gel (KY gel da Johnson & Johnson^®^), and inserted into the vaginal cavity. Then the participant was asked to perform three maximal voluntary PFM contractions, recorded for 15 seconds, with a 3-minute rest period after each one of them. The following verbal command was given: “When I ask you, please perform a pelvic floor contraction as hard as possible and maintain it as long as you can, then relax when you get tired”^13^.

It is worth noting that, the vaginal dynamometer was calibrated by the manufacturer and subsequently tested by the researchers in a previous intra- and inter-rater reliability study^13^, demonstrating a good (i.e. >0.75) Intraclass Correlation Coefficient (ICC) for all analyzed dynamometric measurements (maximum strength, average strength, and endurance).

### Interventions

Two different training protocols were used to investigate and compare their effects on PFM strength:

### APT_VR protocol

**Figure 2:**
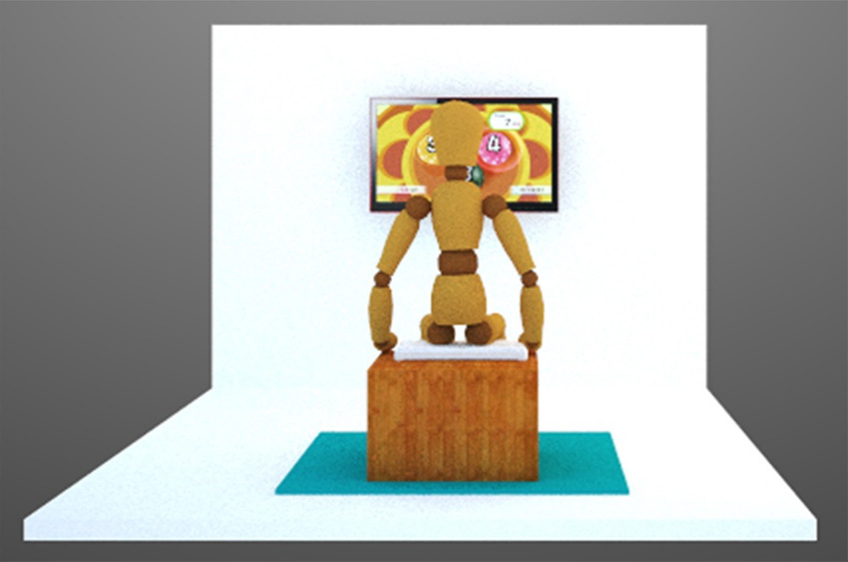
The participant’s position during abdominopelvic training by virtual reality. The virtual reality exercises were carried out, focusing on the abdominopelvic cavity, while sitting on a Wii Balance Board*™*. The participant was advised to perform different exercises using pelvic movements (anteversion, retroversion, lateral tilt, and circumduction), as well as, gently, contract the lower abdominal region (transversus abdominis muscle).

A specific virtual reality protocol was developed by the researchers^14^ through virtual games which promote exercises focusing on the abdominopelvic cavity, using as a therapeutic means a *Wii™* console with a *Wii Fit Plus™* CD game, from which the following sub-games were selected: *Lotus Focus™*, *Penguin Slide™*, *Table Tilt™*, and *Balance Bubble™*. The protocol was designed for the participant to play the games while sitting on a *Wii Balance Board™* (Figure 2), performing different exercises using pelvic movements (anteversion, retroversion, lateral tilt, and circumduction), maintaining trunk control and stabilization, together with a mild activation of the abdominal muscles, especially the TrA. All ten sessions followed the same protocol, with the same sequence and duration of games. The *Lotus Focus™* game was carried out at the beginning and at the end of each session followed by *Penguin Slide™*, *Table Tilt™*, and *Balance Bubble™.* The duration of each game was five minutes with a 90-second interval between games, knowing that the number of restarts of the same game varied according to the participant’s level of performance within the same stipulated five minutes. After finishing the sequence of virtual games, a series of abdominopelvic and lower limb muscle stretching was performed. It is worth mentioning that, while the games were carried out, no verbal commands for direct PFM contractions were given by the researcher. However, it is very likely that the PFM were indirectly recruited through the abdominopelvic movements needed during each game.

Before starting the first training session, the participants were taught how to perform these pelvic movements correctly and were advised to activate the lower abdominal muscles alone, especially the TrA^15^ ^-^ ^18^, while playing the virtual games. Thus, throughout the sessions, the participant was reminded to contract the lower abdominal region gently with the following command: “Tighten your abdominal muscles and pull your belly button inward”.

### PFMT_GB protocol

Marques et al.^4^ proposed this protocol that consists of several exercises focusing on the abdominopelvic cavity using a gym ball as a therapeutic means. The gym ball is an exercise tool that is widely used by physical therapists as a body movement facilitator. Pelvic mobility, stretching, strengthening, and relaxation exercises were performed in all sessions in five different positions (supine, followed by sitting on the floor, then on the gym ball, squat, and finally standing positions). Each exercise was repeated five times, alternating with PFM contractions which consisted of four series of 10 fast contractions together with four series of 10 sustained contractions, lasting eight seconds each then followed by a 16-second rest interval, maintaining the same positions.

Although both protocols focused on the abdominopelvic region, we did not ask the participants of the APT_VR group to perform active contractions of the PFM, in order to verify if the pelvic movements, combined with gentle lower abdominal muscle contractions, would have similar results to the other group.

Both training sessions were individually supervised by the same physical therapist, and lasted 30 minutes each, twice a week for five consecutive weeks, totaling 10 sessions. The participants were not advised or instructed to perform any home exercises, however they were encouraged at every session to adhere to the protocol and attend all sessions. The participant who missed more than two training sessions would be excluded from the group.

### Dynamometric analysis

The vaginal dynamometry data were analyzed using three parameters^13^:

- Maximum strength: calculating the difference between the highest and lowest (baseline) strength values, provided by the equipment software, in kgf:
- Average strength: a mean value of the strength curve, provided by the equipment software, in kgf.
- Endurance: equal to the length of time, in seconds (s), during which the participant could maintain a contraction above 60% of her maximum strength.

An average value was calculated for each parameter, using the results of the three recorded maximal voluntary PFM contractions.

### Statistical analysis

The data were analyzed in two different forms to verify both results, first including only those who actually concluded the research protocol (per protocol analysis) and then including all those who originally participated in the study even if they had not concluded the protocols (intention-to-treat analysis). In this latter analysis, the values of the subject who dropped out of the study at any moment after the first assessment were assumed to be the same as the post-training assessment values (last-observation-carried-forward imputation method).

The Kolmogorov-Smirnov test was used to investigate data normality and the Chi-Square test was used to investigate sample homogeneity. The Wilcoxon or the paired t tests were used to compare pre and post training time, and the Mann-Whitney test was used for the between-group analysis (APT_VR *versus* PFMT_GB), considering both digital palpation and vaginal dynamometry parameter data. Moreover, the 95% confidence interval (95% CI) was included in the vaginal dynamometry data. The correlation between digital palpation and vaginal dynamometry data was investigated using the Spearman correlation test. The Statistical Package for Social Sciences – SPSS-17.0 program was used, with a significance level of 5%. The effect size and power analysis for the vaginal dynamometry data was performed using the G3 Power software. According to Cohen^19^, the effect size values were divided into “small” (≥20 to <50), “medium” (≥50 to <80), and “large” (≥80).

## Results

The groups were considered homogeneous for both demographic and clinical variables. Of the 60 assessed women, most were white (58.3%), married (58.3%), with complete or incomplete primary education (63.3%), and without any paid labor activity (71.7%). The clinical characteristics of the studied population are described in Table 1.

**Table 1:** The clinical characteristics of the studied population.

	**APT_VR (n=30)**	**PFMT_GB (n=30)**
**Age M(SD)**	61.9 (8.6)	61 (8.5)
**BMI M(SD)**	28.1 (3.9)	28 (3.7)
**Physical activity (f / %)**		
No	9 (30)	9 (30)
Yes (twice per week)	10 (33.3)	8 (26.7)
Yes (3-7 times per week)	11 (36.7)	13 (43.3)
**Urinary incontinence (f / %)**		
Absence	9 (30)	7 (23.3)
SUI symptoms	3 (10)	7 (23.3)
UUI symptoms	8 (26.7)	6 (20)
MUI symptoms	10 (33.3)	10 (33.4)
**Hormonal history**		
**Years of post-menopause M(SD)**	13.3 (8.1)	13 (8.5)
**Hormone replacement (f / %)**		
Have never had	16 (53.3)	16 (53.3)
Were having^*^	4 (13.4)	2 (6.7)
Had previously had	10 (33.3)	12 (40)
**Obstetric history**		
**Parity (f / %)**		
Nulliparous	5 (16.7)	3 (10)
Primiparous/Multiparous	25 (83.3)	27 (90)
**Number of pregnancies M(SD)**	3.5 (2.7)	3.9 (2.9)
**Delivery mode (f / %)**		
Exclusively vaginal	21 (70)	14 (46.7)
Exclusively cesarean	3 (10)	7 (23.3)
Vaginal and cesarean	3 (10)	5 (16.7)
No delivery	3 (10)	4 (13.3)

The data are presented in mean (M), standard deviation (SD) as well as absolute (f) and percent (%) frequencies. Personal, hormonal, and obstetric sample characteristics were presented for both statistical analysis: per-protocol analysis as well as intention-to-treat analysis. BMI: Body Mass Index; APT_VR: Abdominopelvic training by virtual reality; PFMT_GB: Pelvic floor muscle training using a gym ball; SUI: Stress urinary incontinence; UUI: Urgency urinary incontinence; MUI: Mixed urinary incontinence.

* Hormone replacement therapy that has been stable for at least six consecutive months.

Both digital palpation and vaginal dynamometry parameters were analyzed before and after the trainings, using both the per-protocol analysis as well as the intention **-** to-treat analysis. Afterwards, these results were compared between the groups, as presented in Tables 2 and 3.

**Table 2:** Pelvic floor muscle assessment by digital palpation, comparing pre and post training time as well as between groups, using both the per-protocol analysis as well as the intention-to-treat analysis.

	**DIGITAL PALPATION**				
		**Pre-training**	**Post-training**	**Time** **p-value** 1	**Between –Group differences** **p-value** 2
**Per-protocol analysis**	**APT_VR (n=27) f (%)**			**0.0001** ^*^	0.7
	0	0 (0)	0 (0)		
	1	1 (4)	0 (0)		
	2	17 (63)	8 (30)		
	3	8 (30)	15 (56)		
	4	0 (0)	3 (11)		
	5	1 (4)	1 (4)		
	**PFMT_GB (n=20) f (%)**			**0.001** ^*^	
	0	0 (0)	0 (0)		
	1	4 (20)	1 (5)		
	2	6 (30)	6 (30)		
	3	7 (35)	4 (20)		
	4	3 (15)	8 (40)		
	5	0 (0)	1 (5)		
**Intention-to-treat analysis**	**APT_VR (n=30) f (%)**			**0.0001** ^*^	0.7
	0	0 (0)	0 (0)		
	1	2 (7)	1 (3)		
	2	18 (60)	9 (30)		
	3	8 (27)	15 (50)		
	4	1 (3)	4 (14)		
	5	1 (3)	1 (3)		
	**PFMT_GB (n=30) f (%)**			**0.0005** ^*^	
	0	0 (0)	0 (0)		
	1	5 (17)	2 (7)		
	2	11 (36)	11 (37)		
	3	9 (30)	6 (20)		
	4	5 (17)	10 (33)		
	5	0 (0)	1 (3)		

The table presents the evaluation and reevaluation times (pre and post-training evaluation) as well as a comparison between the studied groups (APT_VR *versus* PFMT_GB), using both the per-protocol analysis as well as the intention-to-treat analysis. The data were presented in absolute (f) and percent (%) frequencies. APT_VR: Abdominopelvic training by virtual reality; PFMT_GB: Pelvic floor muscle training using a gym ball.

1 Wilcoxon Test.

2 Mann-Whitney Test.

* p≤0.05.

**Table 3:** Pelvic floor muscle assessment by vaginal dynamometry, comparing pre- and post-training time as well as between groups, using both the per-protocol analysis and the intention-to-treat analysis.

		**Within-group analysis**						**Between-group analysis**				
		**Pre-training**	**Post-training**	***p-value*** 1,2	***Power***	***Effect size***	**Between-group mean differences**		**95% CI**	***p-value*** 3	***Power***	***Effect size***
**Per-protocol analysis**	**APT_VR (n=20)**						–0.08 (MS) 0.01 (AS) 1.83 (E)		–0.25 – 0.08 (MS) –0.05 – 0.09 (AS) 0.61 – 3.05 (E)	0.1(*MS*) 0.6 (*AS*) **0.007** ^*^(E)	0.68 (MS) 0.60 (AS) 0.93 (E)	0.32 (MS) 0.17 (AS) 1.04 (E)
	Maximum strength (*Kgf*) M(SD)	0.58 (0.4)	0.67 (0.6)	0.1	0.29	0.17						
	Average strength (*Kgf*) M(SD)	0.23 (0.2)	0.28 (0.2)	**0.02** ^*^	0.36	0.24						
	Endurance (*seconds*) M(SD)	3.12 (1.7)	4.03 (2.4)	**0.05** ^*^	0.57	0.42						
	**PFMT_GB (n=15)**											
	Maximum strength (*Kgf*) M(SD)	0.71 (0.3)	0.89 (0.4)	**0.02** ^*^	0.74	0.46						
	Average strength (*Kgf*) M(SD)	0.32 (0.1)	0.34 (0.2)	0.5	0.52	0.11						
	Endurance (*seconds*) M(SD)	4.27 (2.1)	3.35 (1.7)	**0.04** ^*^	0.75	0.48						
**Intention-to-treat analysis**	**APT_VR (n=23)**						–0.03 (MS) 0.02 (AS) 1.37 (E)		–0.16 – 0.09 (MS) –0.03 – 0.07 (AS) 0.46 – 2.28 (E)	0.5 (*MS*) 0.3 (*AS*) **0.003** ^*^ **(E)**	0.52 (MS) 0.6 (AS) 0.9 (E)	0.14 (MS) 0.31(AS) 0.89 (E)
	Maximum strength (*Kgf*) M(SD)	0.57 (0.4)	0.65 (0.5)	0.1	0.56	0.16						
	Average strength (*Kgf*) M(SD)	0.2 (0.1)	0.3 (0.2)	**0.02** ^*^	0.6	0.21						
	Endurance (*seconds*) M(SD)	2.93 (1.7)	3.73 (2.4)	**0.05** ^*^	0.73	0.37						
	**PFMT_GB (n=24)**											
	Maximum strength (*Kgf*) M(SD)	0.63 (0.3)	0.74 (0.4)	**0.02** ^*^	0.67	0.29						
	Average strength (*Kgf*) M(SD)	0.3 (0.1)	0.3 (0.1)	0.5	0.51	0.06						
	Endurance (*seconds*) M(SD)	4.12 (2.1)	3.54 (1.9)	**0.04** ^*^	0.67	0.28						

The table presents the evaluation and reevaluation times (pre and post-training evaluation) as well as a comparison between the studied groups (APT_VR *versus* PFMT_GB), using both the *per-protocol analysis* as well as the *intention-to-treat analysis*. The data were presented in mean (M) and standard deviation (SD). Note that some participants did not undergo PFM assessment by vaginal dynamometry due to their inability to perform the test due to pain/discomfort when the sensor was introduced. APT_VR: Abdominopelvic training by virtual reality; PFMT_GB: Pelvic floor muscle training using a gym ball; 95% CI: 95% Confidence Interval; Kgf: Kilogram/force; MS: Maximum strength; AS: Average strength; E: Endurance.

1 Wilcoxon Test.

2 Paired t Test.

3 Mann-Whitney Test.

* p≤0.05.

A correlation between the maximum strength dynamometric measurements and digital palpation, considering all of the participants, was observed in both pre (p=0.0001 and r=0.6) and post (p<0.0001 and r=0.8) training periods, according to Spearman‘s correlation coefficient.

## Discussion

Based on the studies of Kegel^20^, who in 1948 prescribed isolated contractions for PFMT, several researchers have been investigating recently the effects of globalized PFMT protocols^21^ ^-^ ^23^. In spite of this, Bø and Herbert^24^, in a published literature review in 2013, reported that there was no evidence at the time as to the effectiveness of alternative exercise regimens.

In the present study, we applied a protocol previously developed by Marques et al.^4^, which combines a gym ball with active PFM contractions verbally commanded by the supervising researcher (PFMT_GB), and compared it with the abdominopelvic training protocol by virtual reality (APT_VR) proposed by us to encourage the performance of abdominopelvic movements through virtual games that did not necessarily require active PFM contractions, this time without any verbal commands.

Although both groups showed significant improvement in PFM strength when assessed by digital palpation (p<0.05), different kinds of PFM strength improvements were observed for each group while analyzing the vaginal dynamometer data, which can be reflected in a peculiar way in the PFM function. Accordingly, the APT_VR group showed a significant increase in the “average strength” and “endurance” parameters, which possibly demonstrates an improvement in muscular strength maintenance; while the PFMT_GB group showed an increase in the “maximum strength” parameter, which could refer to the power and ability to perform fast contractions. Thus, it allowed us to verify the effect of the training protocols on PFM strength and functionality.

Only the endurance parameter showed a significant difference between groups, given that the APT_VR group had a significant improvement after training, while the PFMT_GB group had a significant decrease in the same parameter after training.

The effectiveness of training protocols that use exclusively abdominal muscle contractions for PFMT is still controversial. Some authors^7^ ^,^ ^21^ describe that training only becomes effective when it is combined with a simultaneous PFM contraction. On the other hand, other authors^17^ encourage TrA training for women who do not have PFM consciousness and awareness before developing the suitable PFMT for improving PFM strength and coordination, due to the synergistic action between the lower abdominal muscles and the PFM.

Since no previous studies were found to explain and justify our findings, we hypothesized that the improvement in maximum strength was achieved after carrying out the protocol that included commands for active PFM contractions (PFMT_GB protocol), whereas the improvement in endurance could be due to the command for sustained lower abdominal contraction in the virtual reality protocol (APT_VR protocol), which reinforces Sapsford and Hodge’s theory^15^ that the TrA and PFM act as part of an integrated abdominopelvic unit, suggesting that this muscle interaction may have developed a better PFM perception and control.

Literature shows that increasing muscle strength depends on triggering various factors such as perception, control, coordination, and hypertrophy of the muscle fibers, which require time, frequency, and intensity^25^. Nonetheless, there are several different assessment and intervention methods used by researchers, which hampers comparison among the findings^24^.

In fact, based on the studied dynamometric parameters and on the fact that there is no golden standard for PFM assessment, it is believed that these kinds of analyses should be encouraged during the evaluation process. Morin et al.^26^ found a good level of reliability in the test-retest of the PFM speed and endurance dynamometric measures, using the Montreal dynamometer. They highly recommended the inclusion of these parameters to verify the effect of PFMT.

Likewise, little is known about the necessary training parameters for the recovery of muscle function as well as maintenance of continence^2^. Thus, more controlled and randomized trials are still needed to provide evidence of the best kind of training or protocol to achieve these purposes.

Another interesting finding in this study was a dropout rate in the APT_VR approximately three times lower than in the PFMT_GB group. This suggests that entertainment exercises are more attractive, facilitate adherence, and motivate the continuation of training, which could help maintain the gains achieved during treatment. Thus, stimulating, interactive, and easily-reproduced PFMT protocols should be encouraged, since the success rate after the intervention protocols depends on the adherence as well as maintenance of the proposed exercises^27^ ^,^ ^28^.

Few studies show the long-term effects of PFMT. According to Bø and Hilde^28^, the chances of maintaining the gains through training range from 41 to 85% and depend on the success rate achieved in the short run. However, Quartly et al.^29^ reported that there is a loss of adherence in the long-term in conventional PFMT programs, such as the Kegel^20^ exercises.

The training protocols performed in this study were designed to investigate the effects on PFM strength after 10 therapy sessions in order to investigate early signs of improvement that indicate the correct direction of the treatment. This time interval has been used in clinical practice as the sufficient time to be able to observe if any neuromuscular adaptations had already occurred, which could lead to long-term effectiveness of the proposed treatment.

Despite the fact that these protocols have already showed significant results regarding PFM strength, 10 training sessions are not enough to develop muscular hypertrophy^25^, hence longer periods of intervention, as well as the verification of their effects on urogynecologic symptoms, accompanied by follow-ups, must be encouraged.

The main limitation of this study was that the sample size was not previously calculated; hence, we presented the power and effect size data, to strengthen the achieved results. Moreover, there was a large loss of follow up in the PFMT_GB group (approximately 33%, n=10), and even using the intention-to-treat analysis to minimize the bias that this loss caused to the study protocol as well as for the estimation of training effect^30^, this remains an important limitation of the study.

Another limitation of this study was regarding the vaginal dynamometer equipment. Besides measuring only anteroposterior unidirectional compressive strength, it is worth noting that, some participants did not undergo PFM assessment by vaginal dynamometry due to their inability to perform the test due to pain/discomfort when the sensor was introduced, probably due to a decrease in vaginal elasticity, a characteristic symptom of the postmenopausal period, which limited the study sample.

The focus of this study was to investigate the effect of abdominopelvic training by virtual reality on PFM response in order to conduct a future study on its effect on incontinent women. However, we believe that a morphological parameter analysis could have provided additional information to the studied variables and could have shown the effect of both protocols on the biometric conditions of the PFM.

Further controlled trials with larger sample sizes and using different image methods, including perineal or trans-labial ultrasound, could contribute to the generalization of these findings and clarify the real effects of this virtual reality protocol on PFM function, as well as on urinary incontinence symptoms.

In summary, both training protocols improved the overall PFM contraction. Nevertheless, the abdominopelvic training by virtual reality showed improvement in the capacity to maintain the PFM contraction, i.e. increase in both endurance and average strength. In contrast, pelvic floor muscle training with the gym ball showed an increase in the maximum strength of the PFM contraction with a subsequent decrease in endurance, suggesting that both training protocols can be further explored in clinical research.
